# Building Blocks of 2,3-Diaminopropionic Acid Functionalized with an Oxa-Acid Spacer Shift the Functional Profile of Human Cathelicidin LL-37: Enhanced Enzymatic Stability and Selective Suppression of Neutrophil Degranulation

**DOI:** 10.3390/ph19081152

**Published:** 2026-07-24

**Authors:** Wiktoria Rejmak, Katarzyna Bury, Marta Bauer, Wojciech Kamysz, Magdalena Wysocka, Adam Lesner

**Affiliations:** 1 Faculty of Chemistry, University of Gdańsk, Wita Stwosza 63, 80-308 Gdańsk, Poland; wiktoria.rejmak@phdstud.ug.edu.pl (W.R.); magdalena.wysocka@ug.edu.pl (M.W.); 2 Intercollegiate Faculty of Biotechnology, University of Gdańsk and Medical University of Gdańsk, Antoniego Abrahama 58, 80-307 Gdańsk, Poland; katarzyna.bury@biotech.ug.edu.pl; 3 Faculty of Pharmacy, Medical University of Gdańsk, Al. Gen. J. Hallera 107, 80-416 Gdańsk, Poland; marta.bauer@gumed.edu.pl (M.B.); kamysz@gumed.edu.pl (W.K.)

**Keywords:** cathelicidin LL-37, antimicrobial peptide, DAPEG, 2,3-diaminopropionic acid, proteinase 3, myeloperoxidase, PAD citrullination, solid-phase peptide synthesis, neutrophil degranulation, HL-60

## Abstract

**Background/Objectives:** The human cathelicidin LL-37 (37 residues; net charge +6) couples broad-spectrum antimicrobial activity with immunomodulatory function, but rapid proteolysis and peptidylarginine deiminase (PAD)-mediated citrullination limit its therapeutic use. We aimed to enhance enzymatic stability and to test whether substitution with 2,3-diaminopropionic acid (Dap) building blocks bearing an oxa-acid spacer (DAPEG) shifts the functional profile of the LL-37 scaffold. **Methods:** Six LL-37 analogs were synthesized using Fmoc/tBu solid-phase peptide synthesis, replacing all five arginine, all six lysine, or three isoleucine residues with Dap bearing a one- or two-unit oxa-acid spacer. Identity, purity and secondary structure were assessed via mass spectrometry, ultra-performance liquid chromatography and circular dichroism. Proteolytic stability against human proteinase 3 (PR3), resistance to PAD2/PAD4 citrullination, DNA binding (dynamic light scattering), antibacterial activity, MTT cytotoxicity, and lipopolysaccharide-induced degranulation (PR3 and myeloperoxidase) in differentiated HL-60 cells were evaluated. **Results:** Proteolytic stability against PR3 increased 5.7-fold for the arginine-substituted Dap(GO1) analog, and both arginine analogs resisted PAD2- and PAD4-mediated citrullination, with Dap(GO2) being the most resistant. DNA binding was qualitatively retained, although dynamic light scattering showed weaker complex compaction for lysine analogs. All six analogs lost direct antibacterial activity and were non-cytotoxic at 1–10 µM. In differentiated HL-60 cells, the lysine-substituted Dap(O1) and Dap(O2) analogs suppressed lipopolysaccharide-induced secretion of both PR3 and myeloperoxidase from azurophilic granules, whereas isoleucine analogs were suppressed partially and arginine analogs behaved like native LL-37. **Conclusions:** The DAPEG framework shifts LL-37 from microbicidal toward degranulation-suppressive activity, providing a modular route to tune cathelicidin function. The intracellular mechanism (TLR4/FPR2 engagement, cytokine signaling) was not assayed.

## 1. Introduction

LL-37 is the only cathelicidin family member identified in humans [1]. It is generated by proteolytic processing of the precursor hCAP-18 by proteinase 3 (PR3) in neutrophils and by kallikreins in epithelial cells [2]. The mature peptide—LLGDFFRKSKEKIGKEFKRIVQRIKDFLRNLVPRTES—carries a net positive charge of +6 at physiological pH and adopts an amphipathic α-helix on contact with anionic phospholipid bilayers [3,4], producing membrane disruption through carpet/toroidal-pore mechanisms with broad-spectrum activity against Gram-positive and Gram-negative bacteria, fungi and enveloped viruses [5,6,7]. Beyond direct microbicidal activity, LL-37 functions as a multipotent immunomodulator [8]: it chemoattracts neutrophils, monocytes and T cells through the formyl peptide receptor-like 1 (FPRL1/FPR2) [9]; delays constitutive neutrophil apoptosis [10]; modulates toll-like receptor 4 (TLR4) signaling [11,12]; and contributes to neutrophil extracellular trap (NET) formation and degranulation [13,14,15].

Two major obstacles limit clinical translation of LL-37. First, the peptide is rapidly degraded by host and bacterial proteases, with a biological half-life in serum and wound fluid of minutes to hours [2,7,16]. Second, peptidylarginine deiminases (PAD2, PAD4), activated by intracellular calcium during NETosis [13], citrullinate the arginine side chains of LL-37, eliminating its positive charge and abolishing membrane-disrupting activity while conferring autoantigenic properties implicated in psoriasis and systemic lupus erythematosus [17,18,19]. Multiple chemical strategies have been pursued to overcome these limitations, with varying success in balancing stability, activity and selectivity [20]. These include selective amino-acid substitution [21], hydrocarbon stapling [22], N-methylation [23], engineered SAAP-148 [24], PEGylation of the N-terminus or specific side chains [16], D-amino-acid scanning to produce protease-resistant enantiomers [25], cyclization to constrain the active conformation [26], self-assembly into functional supramolecular structures [27], and encapsulation in lipid nanoparticles or polymeric micelles for stabilized delivery [28]. While each strategy has demonstrated partial success in preserving antibacterial potency, none has simultaneously addressed proteolytic stability, PAD resistance, biocompatibility and tunable immunomodulatory function.

An alternative strategy that has emerged from the work of Wysocka and colleagues uses building blocks based on 2,3-diaminopropionic acid (Dap) modified on the side-chain amino group with a functionalized oxa-acid: the DAPEG framework [29,30,31,32]. These bifunctional monomers are fully compatible with Fmoc solid-phase peptide synthesis (SPPS): a guanidinium-bearing oxa-acid converts Dap into an Arg mimetic, while an amino-bearing oxa-acid converts Dap into a Lys mimetic. The hydrophilic oxa-spacer increases aqueous solubility, suppresses aggregation and provides PEG-like steric shielding [29,30]. DAPEG polymers have recently been shown to penetrate mammalian cell membranes without cytotoxicity and to mediate transfection [31,32], establishing the platform as biocompatible.

Here we report the synthesis and biological characterization of six DAPEG-modified analogs of LL-37 in which (i) all five Arg residues (positions 7, 19, 23, 29, 34), (ii) all six Lys residues (positions 8, 10, 12, 15, 18, 25) or (iii) all three Ile residues (positions 13, 20, 24) were substituted with Dap derivatives bearing either a one-unit or a two-unit oxa-spacer. The aims were to (a) determine whether DAPEG substitution preserves the structural and DNA-binding features of LL-37; (b) quantify protection against proteolytic degradation and against PAD2/PAD4-mediated citrullination; (c) assess cytotoxicity and direct antibacterial activity; and (d) functionally characterize modulation of neutrophil-like cells using differentiated HL-60 cells with LPS challenge, measuring two independent degranulation markers—proteinase 3 (PR3) and myeloperoxidase (MPO)—both co-stored in azurophilic primary granules [15,33]. The concordant readout of two independent granule markers is intended to provide robust evidence for the suppressive phenotype of the most promising analogs.

## 2. Results

### 2.1. Synthesis and Physicochemical Characterization

All six DAPEG analogs and native LL-37 were obtained by Fmoc/tBu SPPS. MALDI-TOF mass spectrometry confirmed identity within 3.5 Da of theoretical values (Table 1). UPLC retention times ranged from 8.06 to 8.52 min. Purification yields after RP-HPLC were 38–45% of the theoretical, consistent with typical performance for 37-residue peptides containing multiple non-canonical residues. A fourth Phe-substituted series (Dap(Z-O1)^5,6,17,27^ LL-37 and Dap(Z-O2)^5,6,17,27^ LL-37) was attempted but did not yield products matching theoretical masses (measured 5176.23 and 5235.18 Da vs. theoretical 5189.88 and 5366.09 Da). These compounds were excluded from biological characterization; their successful synthesis requires optimized coupling conditions and is the subject of ongoing work.

### 2.2. Secondary Structure

**Figure 1:**
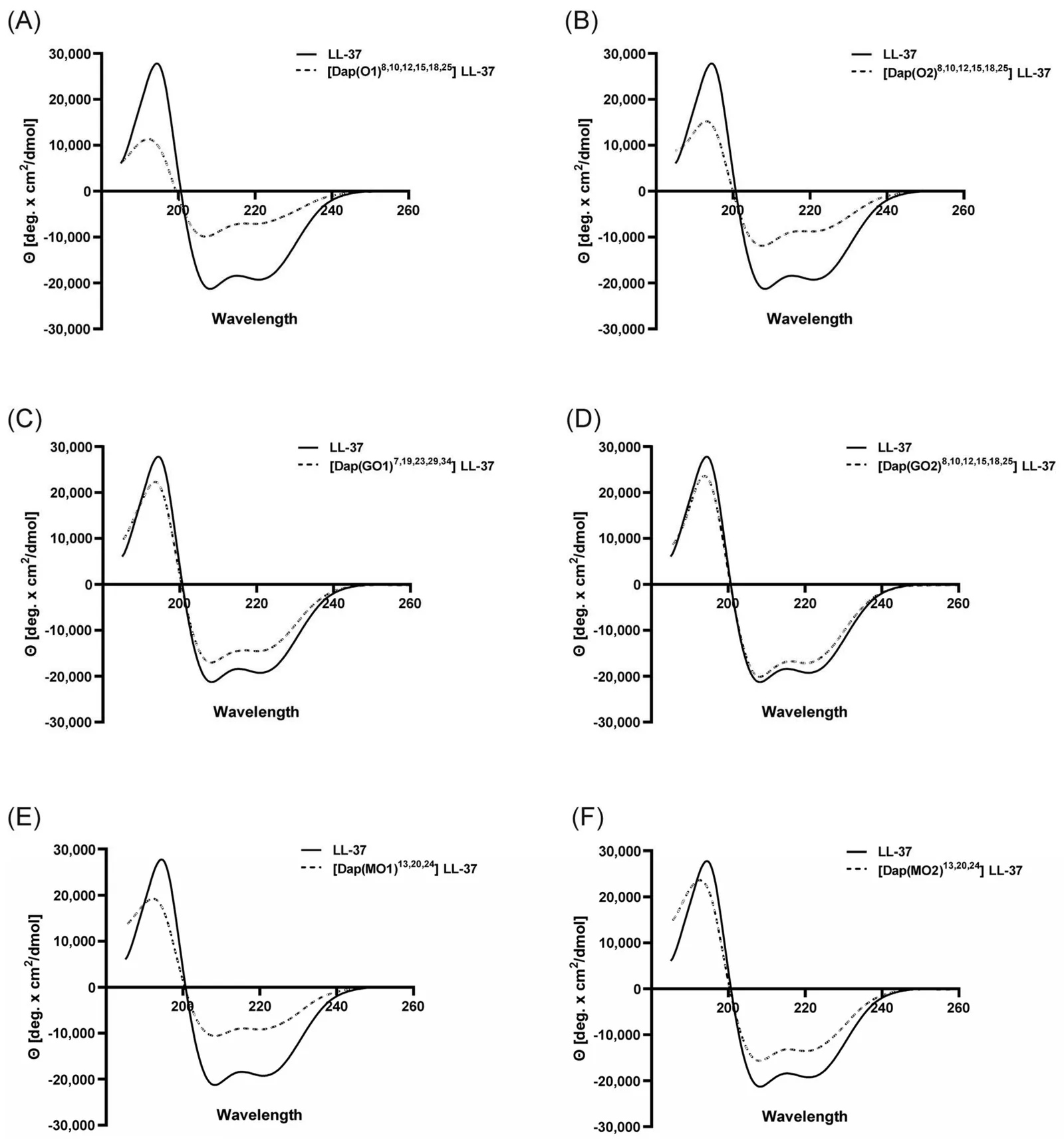
(**A**–**F**) Circular dichroism spectra of LL-37 and the six DAPEG analogs in 50% TFE/water, pH ≈ 7. Each panel overlays the analog (dashed) on native LL-37 (solid). Top row: Dap(O1)^8,10,12,15,18,25^ LL-37 (**left**) and Dap(O2)^8,10,12,15,18,25^ LL-37 (**right**)—substantially reduced helical content. Middle row: Dap(GO1)^7,19,23,29,34^ LL-37 (**left**) and Dap(GO2)^7,19,23,29,34^ LL-37 (**right**)—helical fingerprint preserved. Bottom row: Dap(MO1)^13,20,24^ LL-37 (**left**) and Dap(MO2)^13,20,24^ LL-37 (**right**)—intermediate, partial helix destabilization. *Y*-axis: Θ [deg × cm^2^/dmol]; *X*-axis: wavelength [nm]. Legacy bracket notation in figure labels (drawn from the source thesis) corresponds to the no-bracket notation used in the main text.

Circular dichroism spectra in 50% TFE/water at pH ≈ 7 (Figure 1) revealed three distinct profiles. The arginine-substituted Dap(GO1)^7,19,23,29,34^ LL-37 and Dap(GO2)^7,19,23,29,34^ LL-37 analogs yielded spectra closely matching native LL-37 with characteristic minima near 208 and 222 nm and comparable signal intensity, confirming that the guanidinium-bearing oxa-acid side chain preserves the helix-stabilizing contribution of arginine. The isoleucine-substituted Dap(MO1)^13,20,24^ LL-37 and Dap(MO2)^13,20,24^ LL-37 analogs showed slightly reduced minimum intensities, consistent with partial helix destabilization. The lysine-substituted Dap(O1)^8,10,12,15,18,25^ LL-37 and Dap(O2)^8,10,12,15,18,25^ LL-37 analogs retained the helical fingerprint but with markedly reduced signal intensity, indicating substantially lower helical content—the expected consequence of replacing six cationic Lys residues with neutral oxa-acid side chains and reducing the driving force for helix induction at the membrane interface [5,6,21].

### 2.3. Proteolytic Stability Against Proteinase 3

Resistance to PR3-mediated degradation was strongly dependent on both modification site and oxa-spacer length (Table 2, Supplementary Figure S1). Native LL-37 was rapidly degraded (T½ = 130.2 min), primarily through cleavage at the Val21–Gln22 bond, generating fragments of 2537.24 Da and 2183.32 Da. The Dap(GO1)^7,19,23,29,34^ LL-37 analog exhibited the strongest stabilization—T½ = 740.4 min, a 5.7-fold improvement—with slow, nearly linear degradation over 24 h. By contrast, the longer-spacer Dap(GO2)^7,19,23,29,34^ LL-37 analog (T½ = 120.4 min) was marginally less stable than native LL-37, and only a single hydrolysis fragment exceeded the LC-MS detection threshold; the complementary fragment likely fell below the sensitivity of the method or was lost through coelution with matrix peaks, an interpretation supported by the rapid total decay observed in the kinetic trace (Supplementary Figure S1). The lysine-substituted Dap(O1)^8,10,12,15,18,25^ LL-37 (T½ = 242.3 min) and Dap(O2)^8,10,12,15,18,25^ LL-37 (T½ = 306.2 min) showed intermediate 1.9- and 2.4-fold improvements. The isoleucine-substituted Dap(MO1)^13,20,24^ LL-37 (T½ = 16.5 min) and Dap(MO2)^13,20,24^ LL-37 (T½ = 44.4 min) displayed reduced stability relative to native LL-37, indicating that DAPEG substitution at Ile positions actually destabilizes the central helix region against PR3 attack.

### 2.4. Resistance to PAD2- and PAD4-Mediated Citrullination

Citrullination of arginine residues was quantified by LC-MS as +1 Da mass shifts (Table 3). Native LL-37 underwent a cumulative mass increase of approximately +4 Da, first detectable at 2 h and reaching its maximum by 6 h with both PAD2 and PAD4, consistent with conversion of several of its five arginines. The Dap(GO1)^7,19,23,29,34^ LL-37 analog showed delayed conversion: the first +1 Da shift appeared only at 4 h, and the total shift at 24 h corresponded to approximately +2 Da. The Dap(GO2)^7,19,23,29,34^ LL-37 analog conferred the highest resistance: the first +1 Da shift appeared only at 6 h, and the total shift at 24 h corresponded to citrullination of just a single side chain. The longer two-unit oxa-spacer thus provides superior steric shielding of the guanidinium group from the PAD active site, despite the same spacer giving inferior PR3 protection (Section 2.3). This position- and target-specific dependence on spacer length is mechanistically informative and discussed in Section 3.3.

### 2.5. DNA-Binding Capacity

**Figure 2:**
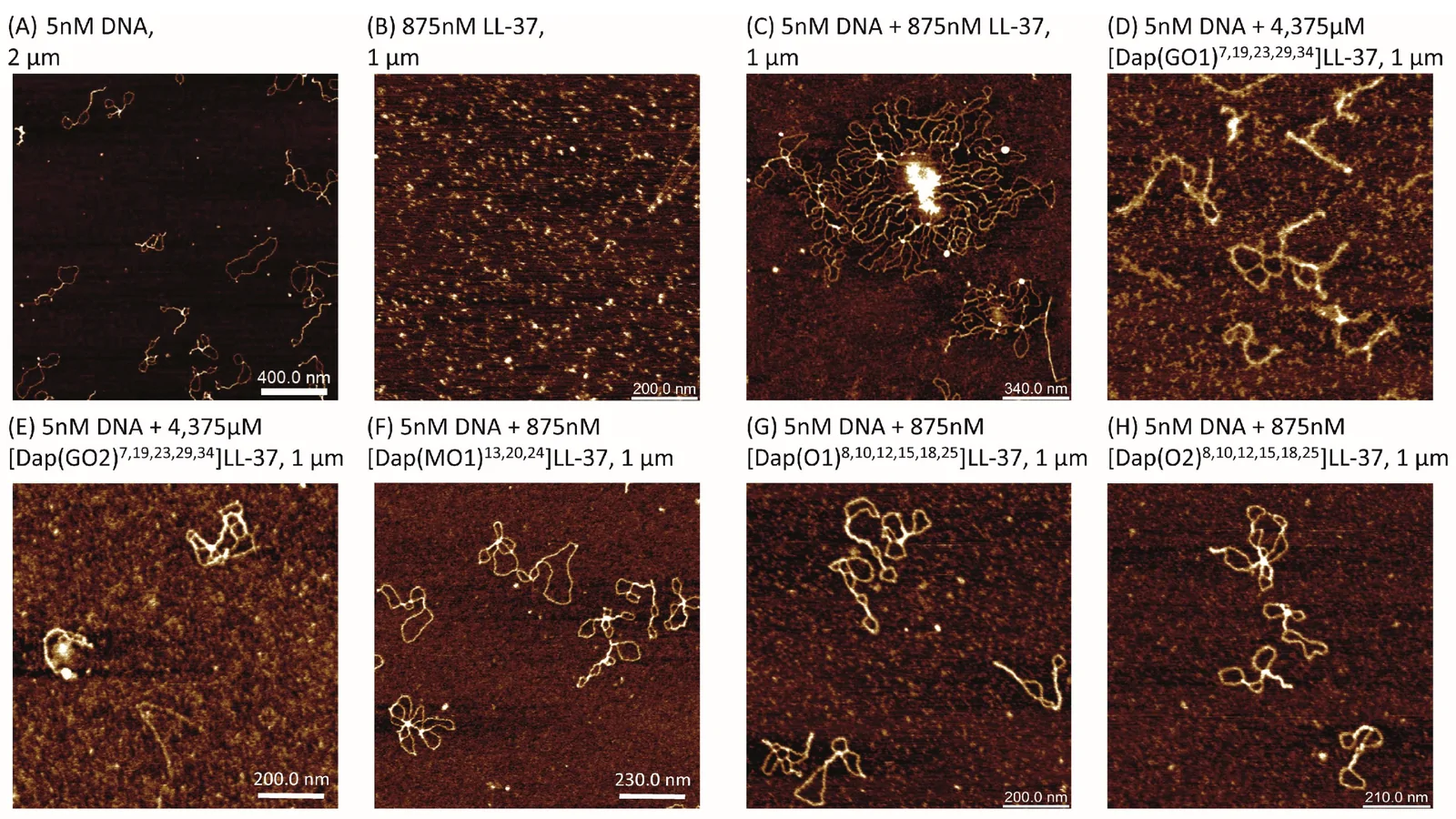
AFM imaging of peptide–pUC19 complexes (N/P = 0.2:1, 8 mM MgCl_2_, 5 nM pUC19, 0.875 nM peptide unless otherwise indicated). (**A**) pUC19 alone (scale 400 nm); (**B**) LL-37 alone (200 nm); (**C**) LL-37 + pUC19 (340 nm); (**D**) Dap(GO1)^7,19,23,29,34^ LL-37 + pUC19 (at 4.375 µM peptide, fivefold higher than the other analogs to compensate for the lower effective cationic load of the guanidinium-bearing DAPEG side chain; 200 nm); (**E**) Dap(GO2)^7,19,23,29,34^ LL-37 + pUC19 (at 4.375 µM peptide; 200 nm); (**F**) Dap(MO1)^13,20,24^ LL-37 + pUC19 (230 nm); (**G**) Dap(O1)^8,10,12,15,18,25^ LL-37 + pUC19 (200 nm); (**H**) Dap(O2)^8,10,12,15,18,25^ LL-37 + pUC19 (210 nm). The Dap(MO2)^13,20,24^ LL-37 panel is omitted because the corresponding AFM image did not meet the resolution criteria applied to the other panels; DLS data for this analog are reported in Figure 3.

**Figure 3:**
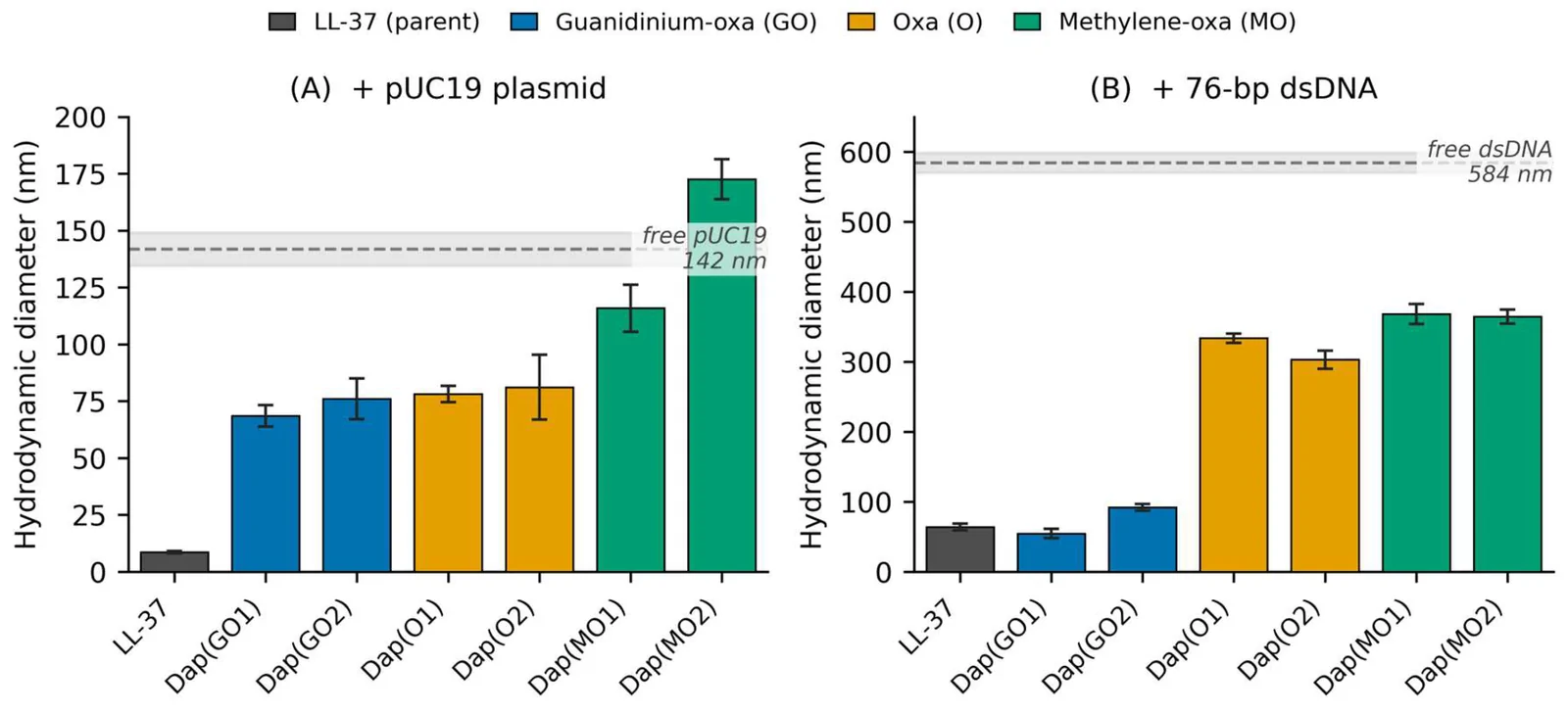
DLS particle-size measurements of peptidomimetics and their complexes. Left panel: complexes with pUC19 plasmid (2688 bp); right panel: complexes with 76-bp dsDNA. *Y*-axis: hydrodynamic diameter (nm); *X*-axis: composition (compound alone vs. compound + DNA template). Numerical hydrodynamic diameters and polydispersity indices (mean ± SD, *n* = 3 independent DLS measurements) are tabulated in Supplementary Table S3. Error bars on the chart represent ± SD (*n* = 3 independent DLS measurements).

All DAPEG analogs retained DNA-binding capacity through qualitative agarose gel electrophoresis and AFM imaging. AFM at N/P = 0.2:1 (Figure 2) showed condensed peptide–pUC19 complexes morphologically similar to those formed by native LL-37, with subtle geometric differences between analogs. For the arginine-substituted analogs Dap(GO1)^7,19,23,29,34^ LL-37 and Dap(GO2)^7,19,23,29,34^ LL-37, the peptide concentration was raised fivefold (to 4.375 µM) to compensate for the reduced effective cationic charge density of the guanidinium-bearing DAPEG side chain relative to native arginine, as detailed in Section 4.5; at the standard 0.875 µM concentration, the complexes formed by these two analogs were below the morphological-detection threshold of the imaging protocol, but they were comparable to the lysine- and isoleucine-substituted analogs once concentration was equalized for the effective cationic load. An AFM panel for Dap(MO2)^13,20,24^ LL-37 is not included in Figure 2 because the available source images did not meet the quality threshold of the rest of the panel; complementary DLS data for this analog are reported in Figure 3 and are consistent with the partial-condensation behavior of the related Dap(MO1)^13,20,24^ LL-37 analog.

MST was attempted but did not yield reliable Kd values: three independent fits for native LL-37 gave widely divergent values (33.8, 96.9 and 62 µM), spanning a 2.9-fold range. This precludes quantitative ranking of analog affinities; resolving it would require an orthogonal method—isothermal titration calorimetry (ITC), biolayer interferometry (BLI), or fluorescence anisotropy—which was not attempted here.

DLS revealed a quantitative gradient not apparent in qualitative assays and provided integration between the two DNA-template models (pUC19 plasmid, 2688 bp; 76-bp dsDNA) needed to interpret the AFM observations (Figure 3, Table S3). Free pUC19 had a hydrodynamic diameter of 141.7 ± 7.2 nm and free 76-bp dsDNA of 584.1 ± 14.3 nm. Three observations dominate. First, for the pUC19 plasmid, native LL-37 produced the most dramatic compaction of any peptide, collapsing the plasmid to 8.6 ± 0.5 nm; all six analogs compacted pUC19 less efficiently—the arginine-substituted Dap(GO1)^7,19,23,29,34^ LL-37 (68.6 ± 4.7 nm) and Dap(GO2)^7,19,23,29,34^ LL-37 (76.0 ± 9.0 nm) and the lysine-substituted Dap(O1)^8,10,12,15,18,25^ LL-37 (78.2 ± 3.6 nm) and Dap(O2)^8,10,12,15,18,25^ LL-37 (81.1 ± 14.2 nm) all condensed the plasmid to ~70–80 nm, while the isoleucine-substituted Dap(MO1)^13,20,24^ LL-37 (115.8 ± 10.4 nm) and Dap(MO2)^13,20,24^ LL-37 (172.5 ± 8.8 nm) compacted it least. Second, the behavior with the short 76-bp duplex was strikingly different and template-dependent: native LL-37 (63.8 ± 4.8 nm), Dap(GO1)^7,19,23,29,34^ LL-37 (54.7 ± 6.6 nm) and Dap(GO2)^7,19,23,29,34^ LL-37 (92.0 ± 4.8 nm) formed small, compact complexes, whereas the lysine-substituted Dap(O1)^8,10,12,15,18,25^ LL-37 (333.7 ± 6.6 nm) and Dap(O2)^8,10,12,15,18,25^ LL-37 (303.1 ± 12.9 nm) and the isoleucine-substituted Dap(MO1)^13,20,24^ LL-37 (368.0 ± 14.3 nm) and Dap(MO2)^13,20,24^ LL-37 (364.5 ± 9.9 nm) formed large assemblies. Third, the contrast between templates resolves the apparent inconsistency in the qualitative data: the Lys- and Ile-substituted analogs compact the supercoiled plasmid reasonably well (~78–173 nm) but fail to compact the short linear duplex (~300–368 nm), whereas the Arg-substituted analogs and native LL-37 compact both templates. This template-length dependence indicates that the topological constraints of the supercoiled plasmid and the short linear duplex are sensed differently depending on the modification class and that reduced cationic charge (Lys → neutral oxa-acid) most strongly impairs compaction of the short, rigid duplex. Quantitative ranking of binding affinities was not possible because of the MST limitations described above.

### 2.6. Antibacterial Activity

MIC assays against three reference bacterial strains revealed a striking and uniform result (Table 4): only native LL-37 retained antibacterial activity, with MIC values within the range 2–8 µM across the three reference strains (typical of LL-37 in CLSI broth microdilution conditions [5,7,21]). All six DAPEG analogs were inactive at concentrations up to 64 µM against *E. coli* ATCC 25922, S. aureus ATCC 25923 and P. aeruginosa ATCC 27853. The complete loss of direct antibacterial activity across all modification classes—including the helix-preserving Arg-substituted analogs—indicates that the DAPEG modifications, despite leaving secondary structure largely intact, disrupt the productive membrane-disruption capacity of the LL-37 scaffold. This finding fundamentally reframes the DAPEG strategy: the modifications do not improve LL-37 as an antibacterial peptide but shift its functional emphasis from microbicidal toward degranulation-suppressive activity (Section 3.2).

### 2.7. Cytotoxicity

MTT assays at 24 h across five cell systems showed no statistically significant viability reduction at 1–10 µM for any compound, with mean cell viability ranging from 92% to 125% of the untreated control across all combinations and showing no significant reduction in any (Table 5; full mean ± SD data at all tested concentrations, including 20 and 50 µM, are provided in Supplementary Information Table S2). For HDFa fibroblasts the lysine-substituted Dap(O1)^8,10,12,15,18,25^ LL-37/Dap(O2)^8,10,12,15,18,25^ LL-37 and isoleucine-substituted Dap(MO1)^13,20,24^ LL-37/Dap(MO2)^13,20,24^ LL-37 analogs showed no significant viability reduction up to 50 µM, while the arginine-substituted Dap(GO1)^7,19,23,29,34^ LL-37/Dap(GO2)^7,19,23,29,34^ LL-37 analogs reduced HDFa viability to ~50% at 50 µM (*p* < 0.001 vs. control; Table S2). Accordingly, the IC_50_ of the GO analogs against HDFa is ≈50 µM, whereas that of the O and MO analogs remains >50 µM. CRL-1472 bladder carcinoma cells were sensitive at 50 µM to all compounds, as were normal HB2 mammary epithelial cells (≈50% viability reduction for LL-37 and all analogs); this 50 µM sensitivity was therefore not tumor-selective. Undifferentiated HL-60 promyelocytic cells were reduced to ~42% viability at 50 µM by parent LL-37 (*p* < 0.01) but were unaffected by all six analogs (≥96%), indicating that DAPEG substitution abolished the anti-proliferative activity of LL-37 against this leukemic line. In differentiated HL-60 cells, LL-37 and the GO analogs increased viability (up to ~128% at 50 µM)—consistent with the pro-survival effect of LL-37 on neutrophils via PI3K/Akt signaling [10]—the O analogs left viability unchanged, and the MO analogs caused a modest reduction (~84–86%). The combination of complete non-cytotoxicity in normal cells at therapeutically relevant concentrations with absence of antibacterial activity (Section 2.6) defines an unusual but pharmacologically interesting profile: high biocompatibility without direct microbicidal action.

### 2.8. Modulation of Neutrophil-like HL-60 Degranulation: Concordant PR3 and MPO Readouts

The most pharmacologically novel findings of this study were obtained by measuring two independent markers of azurophilic granule release—PR3 and MPO—from the same supernatants of differentiated HL-60 cells exposed to peptides at 5.05 µM (equivalent to 25 µg/mL across all analogs; Section 4.9), with or without subsequent LPS challenge. The two-marker design discriminates between genuine inhibition of degranulation and assay-specific artifacts: concordant suppression of both PR3 and MPO indicates a true effect on granule release, while marker-specific responses would imply alternative mechanisms (e.g., direct enzyme inhibition or selective signaling).

**Figure 4:**
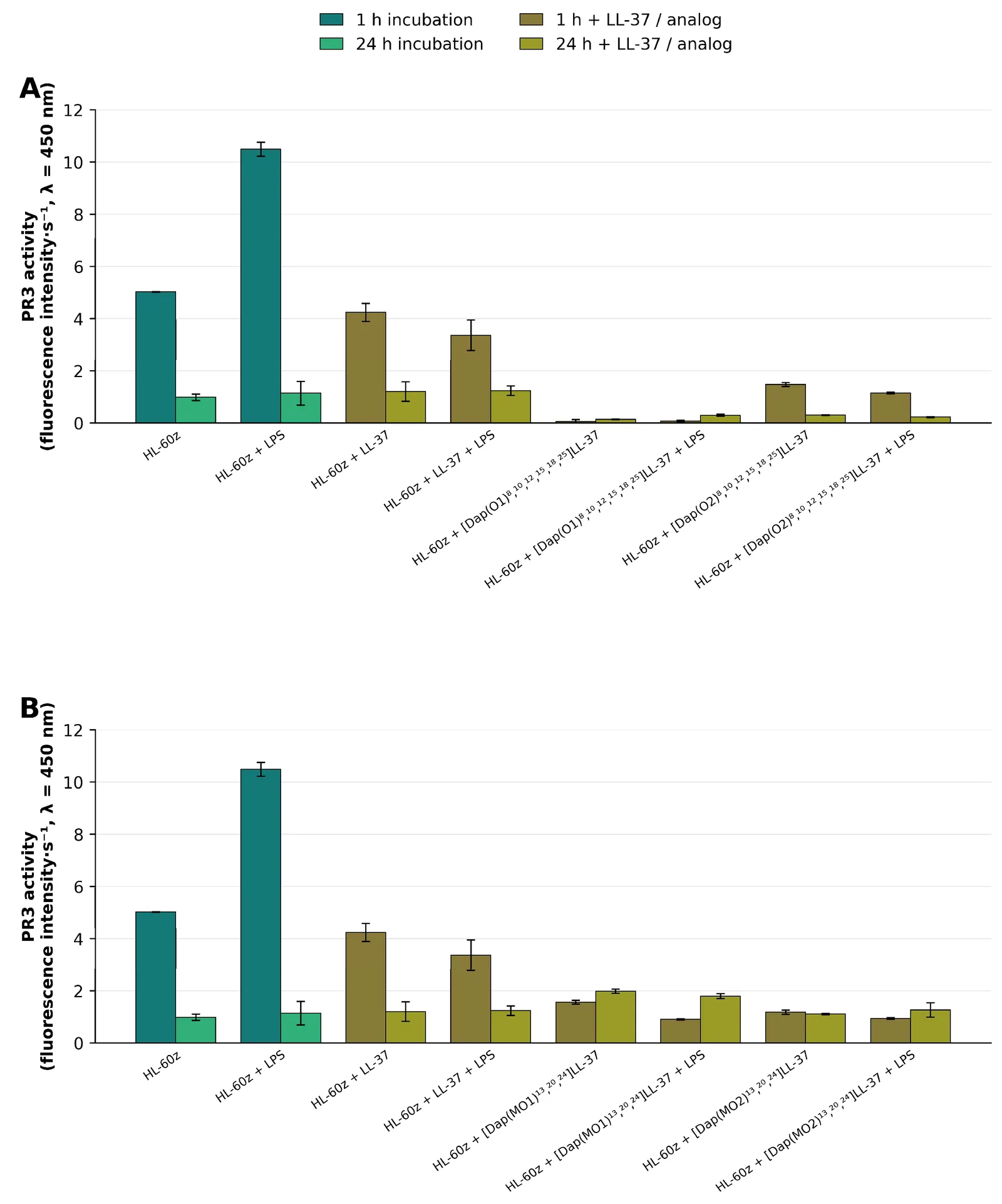

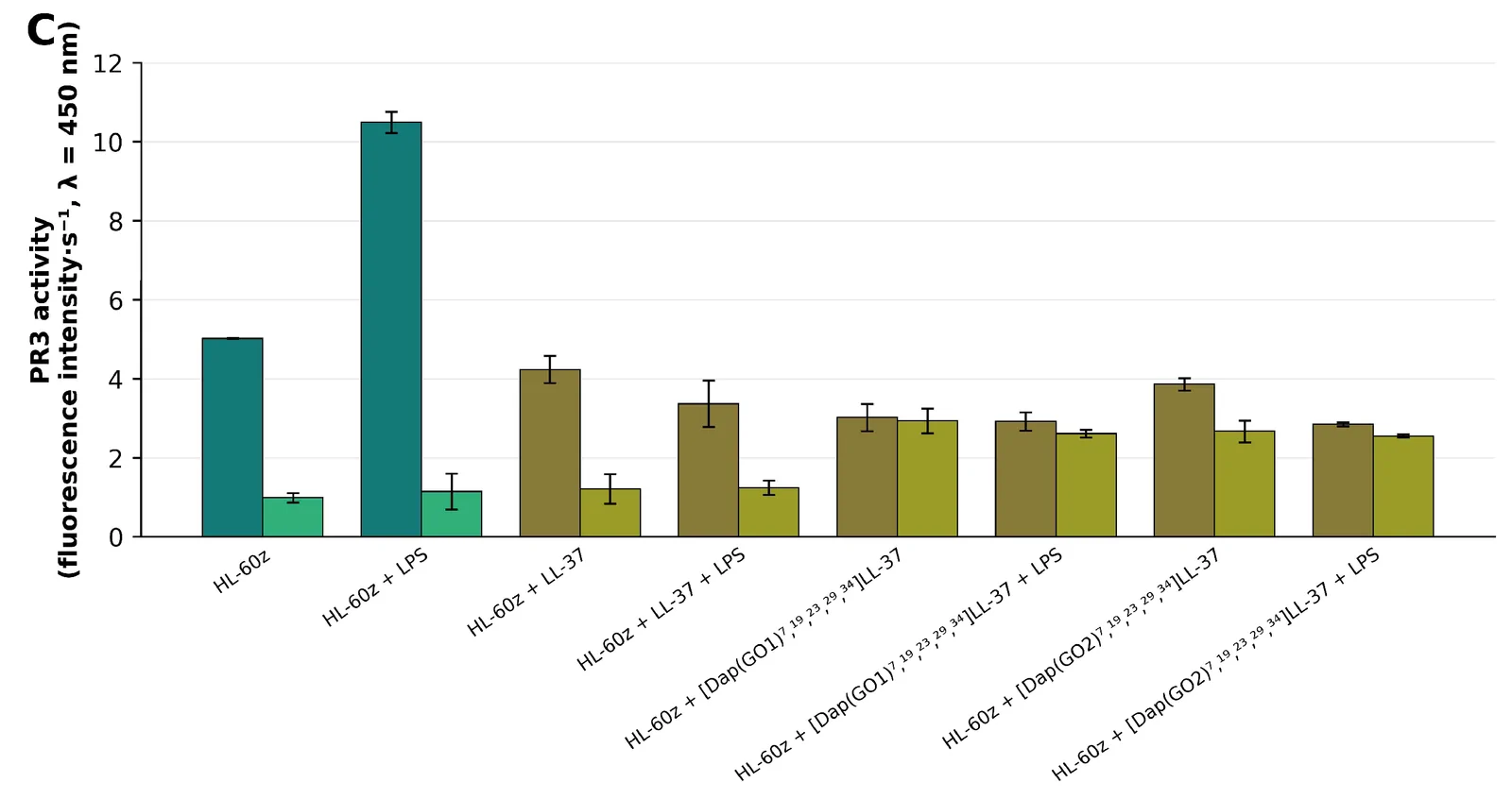
Proteinase 3 secretion from differentiated HL-60 cells exposed to compounds (5.05 µM, equivalent to 25 µg/mL) for 1 h or 24 h, with or without LPS (0.198 µM, 45 min). (**A**) Dap(O1)^8,10,12,15,18,25^ LL-37 and Dap(O2)^8,10,12,15,18,25^ LL-37; (**B**) Dap(MO1)^13,20,24^ LL-37 and Dap(MO2)^13,20,24^ LL-37; (**C**) Dap(GO1)^7,19,23,29,34^ LL-37 and Dap(GO2)^7,19,23,29,34^ LL-37. The four-color palette encodes time × peptide status (1 h vs. 24 h × ± LL-37 analog); LPS condition is indicated separately on the *X*-axis. *Y*-axis: fluorescent intensity/s at λ_em = 450 nm; all three panels were re-plotted with a uniform *Y*-axis label and scale. Mean ± SD, *n* = 3.

**Figure 5:**
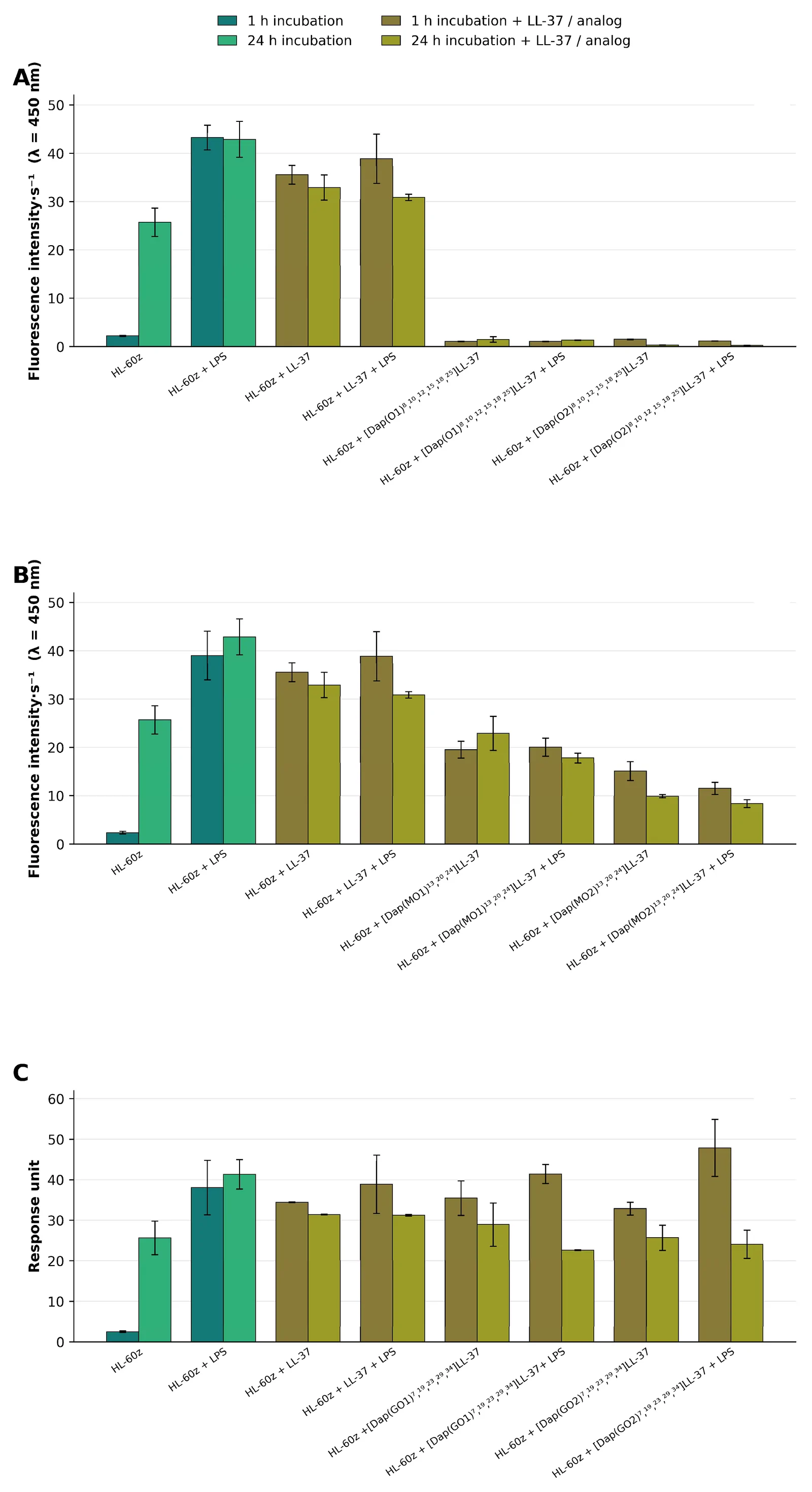
Myeloperoxidase activity in the same supernatants as Figure 4. (**A**) Dap(O1)^8,10,12,15,18,25^ LL-37 and Dap(O2)^8,10,12,15,18,25^ LL-37; (**B**) Dap(MO1)^13,20,24^ LL-37 and Dap(MO2)^13,20,24^ LL-37; (**C**) Dap(GO1)^7,19,23,29,34^ LL-37 and Dap(GO2)^7,19,23,29,34^ LL-37. Concordance with the PR3 pattern across all three panel families (Figure 4) supports the interpretation that the suppression observed for the lysine-substituted analogs is a genuine inhibition of degranulation rather than a marker-specific artifact or direct enzyme inhibition. The *Y*-axis of panel (**C**) reads “response unit” in the source export; the units are identical to those of panels (**A**,**B**) (same plate reader and detection channel). Mean ± SD, (*n* = 3).

**Arginine-substituted analogs (Figure 4C and Figure 5C).** Dap(GO1)^7,19,23,29,34^ LL-37 and Dap(GO2)^7,19,23,29,34^ LL-37 produced PR3 and MPO secretion patterns broadly similar to native LL-37: both compounds activated release at 1 h, with LPS co-stimulation giving the strongest synergistic response. Activation was substantially attenuated at 24 h. The two readouts are concordant, confirming the activating phenotype across both granule markers.

**Lysine-substituted analogs (Figure 4A and Figure 5A)—primary degranulation-suppressive phenotype.** Dap(O1)^8,10,12,15,18,25^ LL-37 and Dap(O2)^8,10,12,15,18,25^ LL-37 did not induce baseline PR3 or MPO release. Crucially, on LPS challenge these analogs substantially suppressed both PR3 and MPO secretion relative to LL-37 + LPS; the suppressive effect was concordant across both markers and persisted at 24 h. This represents the strongest degranulation-suppressive profile observed in this study. The concordance between two mechanistically independent markers stored in the same granule compartment strengthens the conclusion that this is a genuine inhibition of degranulation rather than an assay artifact.

**Isoleucine-substituted analogs (Figure 4B and Figure 5B)—partial suppression.** Dap(MO1)^13,20,24^ LL-37 and Dap(MO2)^13,20,24^ LL-37 showed mild activation alone and partial suppression of LPS-induced PR3 and MPO secretion. The intermediate phenotype is again concordant between the two readouts, confirming a more modest modulatory effect than that seen for the lysine-substituted analogs. A compact summary of all PR3 and MPO data is given in Table 6.

### 2.9. Validation of Assay Specificity Using the PR3-Selective Inhibitor

**Figure 6:**
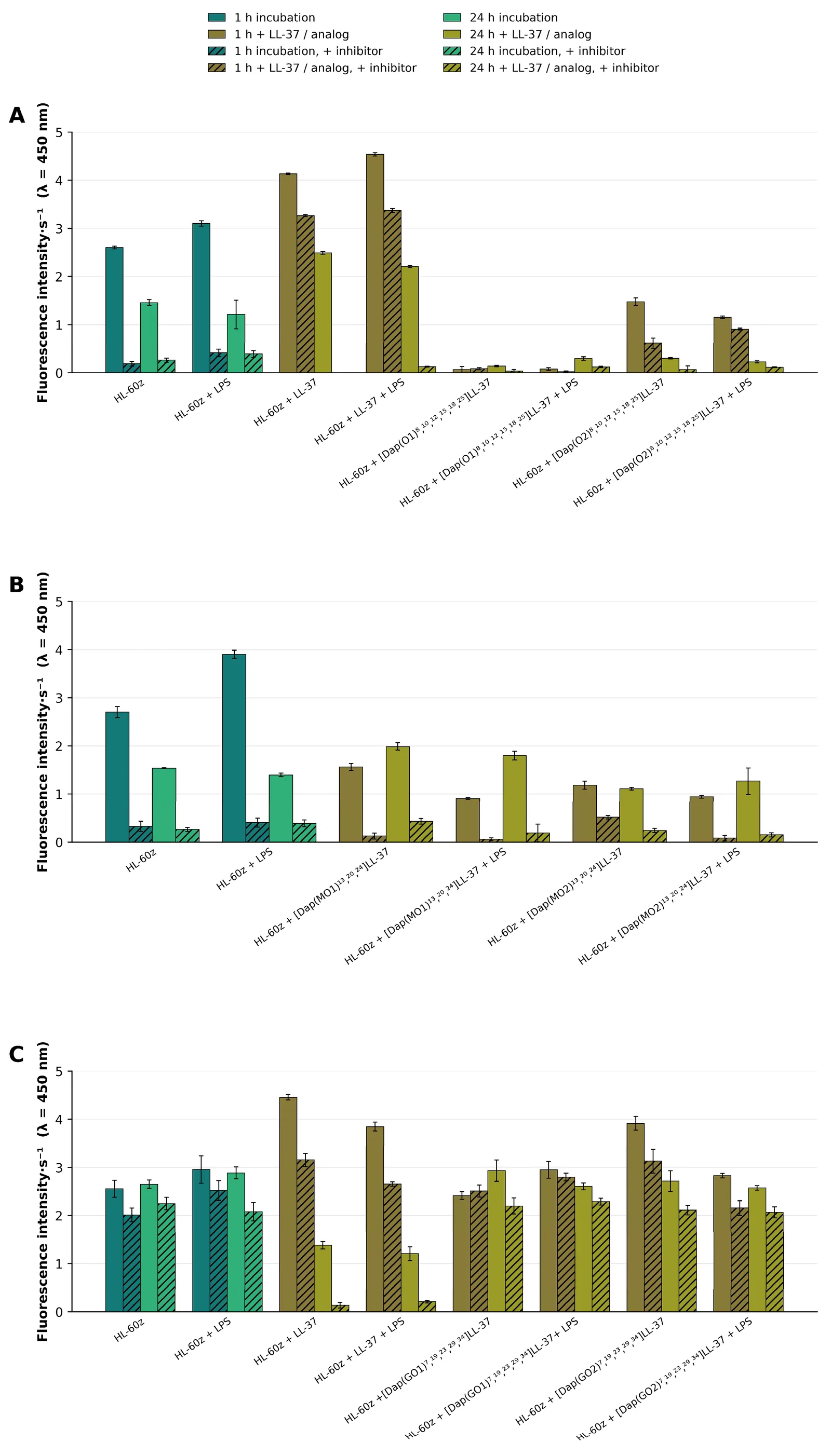
Validation of PR3 assay specificity with the selective phosphonate-based inhibitor Bt-Pro-Tyr-Asp-AbuP(O-C_6_H_4_-4-Cl)_2_ (4.71 × 10^−6^ M, ≈10× molar excess over the IQF substrate). Differentiated HL-60 cells were exposed to compounds (5.05 µM, 1 h or 24 h) with or without LPS challenge, and the supernatants were assayed for PR3 activity in the presence or absence of the inhibitor. (**A**) Oxa analogs Dap(O1)^8,10,12,15,18,25^ LL-37 and Dap(O2)^8,10,12,15,18,25^ LL-37; (**B**) methylene-oxa analogs Dap(MO1)^13,20,24^ LL-37 and Dap(MO2)^13,20,24^ LL-37; (**C**) guanidinium-oxa analogs Dap(GO1)^7,19,23,29,34^ LL-37 and Dap(GO2)^7,19,23,29,34^ LL-37. Solid bars, no inhibitor; hatched bars, + inhibitor. The methylene-oxa experiment (**B**) did not include an LL-37 reference arm. The inhibitor abolished the fluorescent signal across all conditions, confirming that the readout reflects bona fide PR3 active-site catalysis. *Y*-axis: fluorescent intensity/s at λ_em = 450 nm. Mean ± SD (*n* = 3).

To confirm that the fluorescent signal in the PR3 assay reflects bona fide PR3 active-site catalysis rather than non-specific proteolysis or assay artifacts, parallel reactions were performed with addition of the selective phosphonate-based PR3 inhibitor Bt-Pro-Tyr-Asp-AbuP(O-C_6_H_4_-4-Cl)_2_ (4.71 × 10^−6^ M, ≈10-fold molar excess over substrate). As shown in Figure 6, addition of the inhibitor substantially abolished the PR3-derived fluorescent signal across all conditions—baseline HL-60, LPS-stimulated HL-60, and HL-60 exposed to LL-37 or to the oxa, methylene-oxa, and guanidinium-oxa analog pairs (Figure 6A–C, respectively). Importantly, the inhibitor did not abolish the MPO signal measured in parallel from the same supernatants (Figure 5C), confirming that the two-marker concordance described in Section 2.8 reflects two mechanistically independent enzymatic readouts and not cross-reactivity of the detection systems. The residual signal in the (+) inhibitor conditions (≤10% of uninhibited signal in most groups) provides an empirical floor for assay background and supports the conclusion that the suppression seen for the lysine-substituted Dap(O1)^8,10,12,15,18,25^ LL-37 and Dap(O2)^8,10,12,15,18,25^ LL-37 (Figure 4A) is a genuine reduction in PR3 release rather than a methodological artifact.

## 3. Discussion

### 3.1. DAPEG as a Modular Platform for Cathelicidin Engineering

This study demonstrates that the DAPEG framework provides a versatile platform for engineering analogs of LL-37 with tunable enzymatic stability, biocompatibility and degranulation-suppressive function. All six analogs were obtained in moderate but reproducible yields by standard Fmoc SPPS. The same Dap scaffold across all analogs, with functionality varied only by selection of oxa-acid side chain, permits combinatorial exploration of charge, hydrophobicity and helicity through orthogonal substitution at distinct residue classes—a modularity not easily achieved with stapling [22], N-methylation [23], PEGylation [16] or D-amino-acid scanning [25].

### 3.2. Loss of Antimicrobial Activity Despite Preserved Helicity: Implications and Limitations

The complete loss of antibacterial activity across all six DAPEG analogs against tested reference strains is the most consequential structural finding. The loss is uniform across modification classes, and is particularly striking for the arginine-substituted Dap(GO1)^7,19,23,29,34^ LL-37 and Dap(GO2)^7,19,23,29,34^ LL-37 analogs, whose CD spectra closely match that of native LL-37 (Section 2.2). Several non-mutually exclusive mechanisms can account for this paradox. First, CD in 50% TFE/water reports α-helical content in a helix-promoting cosolvent and does not probe the membrane-bound conformation that is the functional state for membrane disruption [3,4,7]; helicity is therefore permissive but not sufficient for productive membrane engagement, and even moderately bulky oxa-acid spacers may disturb the helix–lipid interface enough to abolish disruption while leaving the CD signature intact. Second, the DAPEG side chain displaces the formal positive charge of Arg or Lys onto a remote terminus beyond the hydrophilic oxa-spacer, perturbing the spatial coincidence of cationic and hydrophobic faces required for the carpet/toroidal-pore mechanism [4,5,7]. Third, the oxa-spacer adds a PEG-like steric shield that may reduce insertion into the bilayer interfacial region [29,30].

Direct experimental discrimination among these mechanisms—for example, by membrane depolarization assays in live bacteria, calcein-leakage from negatively charged large unilamellar vesicles (LUVs) mimicking the bacterial inner membrane, N-phenyl-1-naphthylamine (NPN) uptake assays, or solid-state NMR of the analogs in lipid bilayers—was not undertaken in the present study, and the present data therefore do not discriminate among these possibilities. The implication for the present work is that helicity-preservation in CD cannot be taken as evidence of a preserved microbicidal mechanism and that the structure–activity relationship (SAR) conclusion is best framed as a shift of the functional profile of the LL-37 scaffold away from membrane disruption and toward receptor- or LPS-binding-mediated degranulation-suppressive activity. Clinical indications exist where LL-37 immunomodulatory activity is therapeutically desirable without the autoimmune complications of citrullinated LL-37–DNA complexes [17,18,19] and where direct antibacterial activity is neither required nor desired—chronic non-infectious inflammatory disease, immune-mediated wound healing, and sterile inflammation. The DAPEG-modified analogs described here may be more appropriate for such indications than native LL-37 or stapled/SAAP-148-type variants designed to maximize antimicrobial potency [22,24]. We therefore propose that DAPEG modification shifts the functional profile of the LL-37 scaffold—a hypothesis consistent with the biological readouts in this study; the present data do not establish the intracellular mechanism, and the conclusions are limited accordingly.

### 3.3. Position- and Target-Specific Proteolytic and PAD Stabilization

The 5.7-fold protease-stabilization achieved by Dap(GO1)^7,19,23,29,34^ LL-37 is comparable to gains reported for hydrocarbon-stapled [22] and N-methylated [23] LL-37 analogs, but it is obtained without introducing additional hydrophobicity that would elevate hemolytic risk. The non-linear dependence on oxa-spacer length—GO1 > GO2 for PR3 stability; GO2 > GO1 for PAD resistance—is the most mechanistically informative observation. A plausible interpretation is that the shorter GO1 spacer perturbs the Val21 scissile-bond geometry enough to impede PR3 catalysis while still permitting PAD engagement, whereas the longer GO2 spacer accommodates a productive protease conformation but sterically hinders the PAD active site, which is optimized for the natural Arg side chain [13]. The inverted ranking implies that an optimal therapeutic analog may benefit from position-specific combinations of oxa-spacer lengths (GO1 at some Arg positions and GO2 at others), a hypothesis that the present data do not test.

### 3.4. DNA Binding: Coherence and Caveats

All DAPEG analogs retain DNA-binding capacity by qualitative gel-shift and AFM criteria. DLS adds a quantitative, template-dependent layer (Section 2.5, Figure 3). With the supercoiled pUC19 plasmid, native LL-37 is by far the most efficient compactor (8.6 nm), and all six analogs compact the plasmid less efficiently—the Arg- and Lys-substituted analogs to ~70–80 nm and the Ile-substituted analogs least of all. With the short, rigid 76-bp duplex, the pattern inverts for the neutral-charge classes: native LL-37 and the Arg-substituted Dap(GO1)^7,19,23,29,34^ LL-37 and Dap(GO2)^7,19,23,29,34^ LL-37 form small complexes (55–92 nm), whereas the lysine-substituted Dap(O1)^8,10,12,15,18,25^ LL-37 and Dap(O2)^8,10,12,15,18,25^ LL-37 and the isoleucine-substituted Dap(MO1)^13,20,24^ LL-37 and Dap(MO2)^13,20,24^ LL-37 form large assemblies (303–368 nm). The contrast between the two templates resolves the apparent inconsistency a qualitative readout would suggest: reduced cationic charge most strongly impairs compaction of the short linear duplex, while the more topologically constrained plasmid is compacted more uniformly across analogs. The failure of MST to yield reproducible Kd values is a methodological limitation that prevents direct ranking of binding affinities; ranking would require an orthogonal technique (ITC, BLI or fluorescence anisotropy), consistent with the Barron group’s biophysical work on LL-37–nucleic-acid complexation [34].

A pharmacologically intriguing implication of the lysine-substituted profile—DNA-binding with reduced complex compaction, no antibacterial activity, full biocompatibility, suppression of degranulation—is a potential “binding without productive pro-inflammatory signaling” phenotype that may be useful for sequestering self-DNA in autoimmune disease without amplifying inflammation [17,18,19]. The present study does not test this possibility, which would require TLR9 reporter assays in primary plasmacytoid dendritic cells; it is therefore stated as a hypothesis rather than a conclusion.

### 3.5. Selectivity and Biocompatibility

The absence of cytotoxicity at therapeutically relevant concentrations (1–10 µM) across all tested cell lines is notable. Hydrocarbon-stapled and hypercationic LL-37 derivatives frequently show elevated hemolytic activity [21,22]; by contrast, none of the present analogs reduced viability in any cell line up to 20 µM, and against HDFa fibroblasts, the lysine-substituted analogs remained non-cytotoxic even at 50 µM, consistent with their reduced cationicity and reduced non-specific membrane interactions [5,6,21]. At 50 µM, however, viability reduction was not confined to the CRL-1472 bladder carcinoma line: normal HB2 mammary epithelial cells were reduced to a comparable extent (≈50% for LL-37 and all analogs), and the arginine-substituted analogs also reduced HDFa viability. This 50 µM toxicity is therefore not tumor-selective and is more consistent with the general membrane-perturbing activity of LL-37-type peptides at high concentration [7]; the usable concentration window is correspondingly limited to ≤20 µM. The increase in differentiated HL-60 viability across the GO and LL-37 conditions aligns with the anti-apoptotic effect of LL-37 on neutrophils through PI3K/Akt signaling downstream of FPR2 engagement [10].

### 3.6. The Principal Finding: Concordant Suppression of PR3 and MPO Degranulation by Lysine-Substituted Analogs

The defining pharmacological finding of this study is the substantial, concordant suppression of LPS-induced PR3 and MPO secretion by Dap(O1)^8,10,12,15,18,25^ LL-37 and Dap(O2)^8,10,12,15,18,25^ LL-37 in differentiated HL-60 cells. The two-marker design was deliberately introduced to discriminate between genuine inhibition of degranulation and assay-specific artifacts: PR3 and MPO are co-stored in azurophilic primary granules but are detected through mechanistically independent assays (synthetic fluorogenic peptide substrate vs. intrinsic peroxidase activity) [15,33,35]. Concordance between the two readouts therefore provides evidence for an effect on the regulated release of granule contents. Methodological validation using the selective PR3 inhibitor (Figure 6) confirmed that the fluorescent signal reflects bona fide PR3 active-site catalysis and not artifactual fluorescence, further strengthening the interpretation of the suppression phenotype. The magnitude of suppression is large—LPS-induced secretion reduced to levels comparable to vehicle control—and is observed at both 1 h and 24 h.

Several mechanistic hypotheses are consistent with this two-marker concordance and the structural features of the lysine-substituted analogs: (i) reduced helicity and net charge may prevent productive FPR2 engagement while permitting biased signaling that suppresses TLR4 downstream pathways [9,10,11,12]; (ii) the analogs may sequester LPS directly, blocking TLR4 engagement [11,12]; or (iii) they may occupy FPR2 with kinetics that favor a suppressive rather than an activating cascade. The present study does not discriminate among these mechanisms; doing so would require receptor-binding, LPS-binding, TLR4/NF-κB reporter and cytokine assays that lie outside its scope. They are therefore presented as hypotheses, not conclusions. The intermediate phenotype of Dap(MO1)^13,20,24^ LL-37 and Dap(MO2)^13,20,24^ LL-37—partial suppression rather than full inhibition—likely reflects their intermediate structural properties: modestly reduced helicity but full cationic charge from preserved Arg and Lys residues. The dose–response position of MO analogs between the activating GO/Arg-substituted family and the suppressive O/Lys-substituted family confirms that the modulatory phenotype is structurally tunable within the DAPEG framework.

### 3.7. Limitations and Future Directions

Several limitations of this work merit explicit acknowledgement. First, proteolytic stability assays used a single isolated enzyme (PR3); stability in human serum, wound fluid or homogenates of activated neutrophils would more directly reflect translational relevance. Second, the antibacterial assays used three reference strains under standard CLSI broth-microdilution conditions; the uniform inactivity at 64 µM makes recovery of microbicidal function under physiological conditions unlikely, but it cannot be formally excluded. No membrane-level mechanistic validation of the loss of antimicrobial activity (membrane depolarization, LUV calcein leakage, NPN uptake, solid-state NMR) was performed; the geometric, charge-distribution and steric mechanisms outlined in Section 3.2 therefore remain candidate explanations that the present data do not discriminate. Third, the HL-60 differentiation model is a surrogate for primary neutrophils; validation in freshly isolated human peripheral blood neutrophils—with assessment of reactive oxygen species (ROS) burst, NET formation, and chemotactic response in addition to the two granule markers reported here—would be needed to confirm functional translation, which the present data do not establish. Fourth, the present data do not establish a specific intracellular signaling mechanism for the degranulation-suppressive phenotype: TLR4, NF-κB, FPR2 and MAPK pathways were not directly assayed, and no cytokine readouts (TNFα, IL-1β, IL-6, IL-8) were generated; consequently the term “degranulation-suppressive” is preferred throughout to the broader “anti-inflammatory”, which would require positive evidence on multiple signaling axes. Fifth, MST failure to yield reliable Kd values leaves the quantitative DNA-binding ranking unresolved; an orthogonal technique (ITC, BLI or fluorescence anisotropy) would be needed and was not applied here. Sixth, the degranulation-suppressive effect was assessed at a single mass-equivalent concentration (25 µg/mL); the present data establish the effect but do not define its potency (apparent IC_50_) or its concentration dependence. These limitations define the boundaries of the present study and the scope of its conclusions; they are stated so that the findings are not over-interpreted.

## 4. Materials and Methods

### 4.1. Peptide Synthesis

All compounds were synthesized with Fmoc/tBu SPPS on TentaGel S-AC resin pre-loaded with serine (loading 0.24 mmol/g, 90 µm; Iris Biotech GmbH, Marktredwitz, Germany), on a 0.1 mmol scale (0.417 g resin). Backbone assembly was performed on a LibertyBlue microwave synthesizer (CEM Corporation, Matthews, NC, USA) using 0.2 M Fmoc-amino acid in N,N-dimethylformamide (DMF), 0.5 M diisopropylcarbodiimide (DIC) in DMF and 1.0 M Oxyma in DMF (coupling: 165 s at 90 °C). Fmoc deprotection used 20% piperidine/DMF (65 s at 90 °C). At positions of intended modification, Fmoc-Dap(ivDde)-OH was incorporated.

After backbone assembly, ivDde side-chain protecting groups were removed by 2% anhydrous hydrazine in DMF (6 × 15 min). Side-chain acylation with Boc/Pbf-protected oxa-acid derivatives—Boc-Pbf-guanidino-O1Pen-OH (GO1), Boc-Pbf-guanidino-O2Oc-OH (GO2), Boc-O1Pen-OH (O1) or Boc-O2Oc-OH (O2)—was performed in DMF/N-methyl-2-pyrrolidone (NMP)/dichloromethane (DCM) (1:1:1, v/v/v) with trace Triton X-100, activated with DIC and Oxyma, in three sequential cycles at 2×, 1.5× and 1.0× molar excess. Cleavage and global deprotection: trifluoroacetic acid (TFA)/phenol/water/thioanisole/ethanedithiol (82.5/5/5/5/2.5, v/v/v/v/v; 4 h, room temperature). Crude products were precipitated with cold diethyl ether, redissolved in deionized water, lyophilized and purified by reverse-phase high-performance liquid chromatography (RP-HPLC).

### 4.2. Analytical Characterization

Mass: matrix-assisted laser desorption/ionization-time of flight mass spectrometry (MALDI-TOF MS, Bruker Biflex III). Purity: UPLC on Shimadzu Nexera X2 LC-30AD with Phenomenex Aeris Peptide XB-C18 (150 × 2.1 mm, 1.7 µm); gradient 3–90% B over 22 min at 0.4 mL/min. Circular dichroism (CD): 0.2 mg/mL in 50% 2,2,2-trifluoroethanol (TFE)/water, pH ≈ 7. Mass and UPLC measurements served to confirm identity and purity; CD spectra were recorded to assess secondary structure. These determinations were single measurements per compound and are reported without dispersion estimates.

### 4.3. Proteolytic Stability Against Proteinase 3

Reactions: 45 µM peptide and 0.2 µM recombinant human PR3 in 10 mM Tris-HCl/0.01% ammonium acetate, pH 7.5, 37 °C. Aliquots were taken at 0, 1, 2, 4, 6 and 24 h and analyzed using liquid chromatography–mass spectrometry (LC-MS; Bruker HCT Ultra ion trap; Phenomenex Gemini-NX 5µ C18, 4.6 × 150 mm; 10–90% acetonitrile/0.1% formic acid over 50 min). Half-lives were derived from monoexponential decay. Reported half-lives are single determinations from the available source dataset; replicate runs were not available, so per-value standard deviations are not reported.

### 4.4. PAD2 and PAD4 Citrullination Assay

We used PAD2 (3.34 µM) or PAD4 (1.35 µM) with 50 µM peptide in 0.24 M Tris-HCl/0.05 M CaCl2, pH 7.6, 37 °C. Aliquots were taken at 0, 2, 4, 6, 8 and 24 h and analyzed using LC-MS. Each Arg → Cit conversion produces a +1 Da increment. The reported masses are single LC-MS determinations from the available source dataset; replicate runs were not available, so standard deviations are not reported.

### 4.5. DNA-Binding Studies

We performed agarose gel electrophoresis (0.8% agarose, Tris-borate-EDTA (TBE), Midori Green, NIPPON Genetics EUROPE, Düren, Germany) with pUC19 plasmid (2688 bp) at a nitrogen-to-phosphate (N/P) ratio of 1:1 and 5:1, following peptide–DNA complexation protocols established in our laboratory and by Sandgren et al. [36] for LL-37–DNA interaction studies. We performed polyacrylamide gel electrophoresis (PAGE; 4%/8% gels, Tris-acetate-EDTA (TAE)) with synthetic 76-base-pair (bp) double-stranded DNA (dsDNA). We performed microscale thermophoresis (MST; Monolith NT.115, NanoTemper Technologies, Munich, Germany) [37] with Cy5-labeled 76-bp dsDNA (100 nM) and serial peptide dilutions (200 µM starting concentration, 16-fold dilution); three buffer systems were tested (EDBS; PBS/0.05% Tween-20; PBS/0.1% Pluronic F-127) with three independent replicates per condition. We performed atomic force microscopy (AFM) imaging (BioScope Resolve, Bruker, Billerica, MA, USA; ScanAsystFluid+) of peptide–pUC19 complexes (N/P = 0.2:1, 8 mM MgCl2, 5 nM pUC19, 0.875 nM peptide), following established protocols for cationic peptide–DNA complexation imaging [38]; the imaging was performed by Dr. K. Bury (Intercollegiate Faculty of Biotechnology, University of Gdańsk and Medical University of Gdańsk). For Dap(GO1)^7,19,23,29,34^ LL-37 and Dap(GO2)^7,19,23,29,34^ LL-37, the peptide concentration was increased fivefold (to 4.375 µM) to compensate for the reduced effective cationic charge density of the guanidinium-bearing DAPEG side chain relative to native arginine, ensuring comparable complex formation under the same N/P regime. Dynamic light scattering (DLS) and zeta potential measurements were performed on a Litesizer DLS 500 (Anton Paar; Dr B. Szafranek, Physicochemical Measurements Laboratory, Faculty of Chemistry, University of Gdańsk); *n* = 3 measurements per sample.

### 4.6. Antibacterial Activity

We used the minimum inhibitory concentration (MIC) by broth microdilution per Clinical and Laboratory Standards Institute (CLSI) guidelines, with reference strains Escherichia coli ATCC 25922, Staphylococcus aureus ATCC 25923 and Pseudomonas aeruginosa ATCC 27853. The bacterial inoculum was 5 × 10^5^ colony-forming units (CFUs)/mL in cation-adjusted Mueller–Hinton broth. Compounds were tested at 1, 2, 4, 8, 16, 32 and 64 µM for 18–20 h at 37 °C. MIC was defined as the lowest concentration with no visible growth. Each compound was tested in *n* = 3 biological replicates with technical duplicates.

### 4.7. Cell Culture

HB2 (breast epithelial), CRL-1472 (bladder transitional carcinoma stage III), HDFa (primary dermal fibroblasts) and HL-60 (promyelocytic leukemia) cell lines were maintained at 37 °C, 5% CO_2_ in their respective media (full details in Supplementary Table S1). For neutrophil-like differentiation, HL-60 cells were cultured in RPMI-1640 + 10% fetal bovine serum (FBS) + 1% penicillin/streptomycin + 1.25% dimethyl sulfoxide (DMSO) for five days with daily medium exchange and cell count regulation to 10 × 10^6^ cells, following the DMSO-based differentiation protocol established for HL-60-derived neutrophil-like cells [39].

### 4.8. MTT Cytotoxicity Assay

Cells were seeded for 48 h prior to treatment. Compounds were tested at 1, 5, 10, 20 and 50 µM for 24 h. MTT (3-(4,5-dimethylthiazol-2-yl)-2,5-diphenyltetrazolium bromide; 5 mg/mL in PBS) was added and incubated for 4 h; formazan was solubilized in DMSO, with absorbance at 570 nm (SPECTROstar Nano, BMG LabTech, Ortenberg, Germany). *n* = 3 biological replicates with three technical replicates each, and we conducted a one-way ANOVA with Dunnett post hoc vs. untreated control, significance *p* < 0.05. Mean cell viability (% of untreated control) and IC_50_ values where determinable are summarized in Table 5; full mean ± SD data at all tested concentrations are tabulated in Supplementary Information (Table S2).

### 4.9. Neutrophil-like HL-60 Degranulation Assays—Proteinase 3 and Myeloperoxidase

On day 5 of DMSO-induced differentiation, HL-60 cells (5 × 10^6^ cells/well, 0.1% fish gelatin-coated 6-well plates) were exposed to compounds at 5.05 µM for either 1 h or 24 h at 37 °C, followed by LPS challenge (0.198 µM, 45 min). The peptide concentration of 5.05 µM corresponds to a fixed mass concentration of 25 µg/mL across all analogs (peptide MW ≈ 4950 Da), enabling direct comparison on a mass-equivalent basis and reflecting a clinically relevant range for LL-37 [11,12]. The two pre-incubation durations were selected to dissect distinct modes of action: 1 h interrogates acute, membrane- and receptor-proximal effects of the peptide on the degranulation machinery, while 24 h captures prolonged exposure effects encompassing modulation of neutrophil lifespan, gene-expression-dependent changes and slow-developing receptor desensitization that are well established for LL-37 [10,13,40,41]. The 20–24 h pre-incubation followed by LPS challenge corresponds to protocols previously used to study LL-37 effects on primary human polymorphonuclear leukocytes (PMN), in which LL-37 was incubated for 20 h in the presence or absence of *E. coli* LPS and was shown to modulate neutrophil lifespan, granule release and secondary necrosis [40,41]. Extracellular medium was collected. The same supernatants were used for both PR3 and MPO assays to ensure direct comparability of the two markers.

PR3 activity was measured using the internally quenched fluorescent (IQF) substrate ABZ-Tyr-Tyr-Abu-Asn-Glu-Pro-Dap(DNP)-NH_2_ (final concentration 4.73 × 10^−5^ M), in which the ortho-aminobenzoic acid (ABZ) fluorophore is FRET-quenched by the 2,4-dinitrophenyl (DNP) acceptor introduced on the Dap side chain; proteolytic cleavage of the scissile bond between the ABZ donor and DNP acceptor releases the donor fluorescence (excitation maximum λ_ex = 320 nm; emission maximum λ_em = 450 nm). To confirm that the fluorescent signal originated specifically from PR3 activity, parallel reactions were performed with the selective phosphonate-based PR3 inhibitor Bt-Pro-Tyr-Asp-AbuP(O-C_6_H_4_-4-Cl)_2_ (final concentration 4.71 × 10^−6^ M, ≈10-fold molar excess over substrate to ensure complete active-site occupancy). Reactions were conducted in 100 mM Tris-HCl/500 mM NaCl, pH 7.5; fluorescence kinetics were recorded on a CLARIOstar plate reader (BMG LabTech) in continuous kinetic mode at 37 °C. Quantification used the linear portion of the time course; inhibitor-suppressible signal was taken as PR3-specific activity.

MPO activity was measured fluorometrically in the same supernatants using the Myeloperoxidase (MPO) Peroxidation Activity Assay Kit (Fluorometric) (Abcam, Cambridge, UK, ab111749), per the manufacturer’s protocol. *n* = 3 biological replicates per condition with technical duplicates; we performed a two-way ANOVA with Sidak correction for multiple comparisons. Data are shown as mean ± SD.

## 5. Conclusions

The DAPEG framework provides a modular, Fmoc-SPPS-compatible platform for cathelicidin engineering that decouples two of the principal liabilities of native LL-37—proteolytic instability and PAD-mediated citrullination—from each other in a position- and spacer-length-dependent manner. The opposite ranking of GO1 and GO2 against PR3 versus PAD enzymes establishes that proteolytic and post-translational stabilization can be tuned independently within the same chemical framework, which has direct implications for the design of next-generation cathelicidin analogs and is, to our knowledge, the first explicit demonstration of this decoupling within a single peptidomimetic series.

Substitution of arginines with a one-oxa-unit DAPEG block (Dap(GO1)^7,19,23,29,34^ LL-37) yields a 5.7-fold increase in proteolytic half-life against PR3, while substitution with the two-oxa-unit block (Dap(GO2)^7,19,23,29,34^ LL-37) confers the greatest resistance to PAD2- and PAD4-mediated citrullination. Both Arg-substituted analogs lose direct antibacterial activity despite preserved CD-helicity, an outcome that places mechanistic constraints on the role of secondary structure in LL-37 antimicrobial action, and the present antibacterial data do not, on their own, resolve the membrane-level basis of this loss.

The most significant biological outcome of this study is mechanistic and pharmacological rather than antimicrobial: the lysine-substituted Dap(O1)^8,10,12,15,18,25^ LL-37 and Dap(O2)^8,10,12,15,18,25^ LL-37 analogs converted the LL-37 scaffold from an activator into a suppressor of LPS-induced degranulation in differentiated HL-60 cells, as judged by concordant readouts on two mechanistically independent azurophilic granule markers (PR3 and MPO), with retention of DNA-binding capacity and full biocompatibility at therapeutically relevant concentrations. The functional impact of the framework therefore lies not in improved microbicidal potency but in the emergence of a structurally tunable degranulation-suppressive activity that is novel for cathelicidin analogs. Whether this phenotype generalizes to a broader anti-inflammatory profile, and whether it is mediated by TLR4, FPR2, or LPS-sequestration mechanisms, are questions that the present data do not address; the conclusions of this study are therefore limited to the degranulation-suppressive phenotype established by the two independent granule-marker readouts. Within those limits, the DAPEG-modified lysine-substituted analogs warrant prioritization as lead compounds for sterile-inflammation indications.

## Figures and Tables

**Table 1:** Molecular masses (measured and theoretical, MALDI-TOF MS) and UPLC retention times of LL-37 and the six DAPEG analogs.

No.	Compound	Measured MW [Da]	Theoretical MW [Da]	Δ [Da]	tR [min]
1a	Dap(GO1)^7,19,23,29,34^ LL-37	4860.38	4858.60	1.78	8.47
1b	Dap(GO2)^7,19,23,29,34^ LL-37	5079.98	5078.86	1.12	8.06
2a	Dap(O1)^8,10,12,15,18,25^ LL-37	4850.19	4847.41	2.78	8.52
2b	Dap(O2)^8,10,12,15,18,25^ LL-37	5115.05	5111.72	3.33	8.43
3a	Dap(MO1)^13,20,24^ LL-37	4763.05	4760.41	2.64	8.26
3b	Dap(MO2)^13,20,24^ LL-37	4894.99	4892.57	2.42	8.44
LL-37	LLGDFFRKSKEKIGKEFKRIVQRIKDFLRNLVPRTES	4491.34	4492.14	0.80	—

Δ: difference between measured and theoretical mass.

**Table 2:** PR3-mediated proteolysis kinetics.

Compound	Analog MW [Da]	T½ [min]	Fragment 1 MW [Da]	Fragment 2 MW [Da]	Fold vs. LL-37
LL-37	4491.34	130.2	2537.24 (Val21↓)	2183.32	1.0×
Dap(GO1)^7,19,23,29,34^ LL-37	4858.92	740.4	2852.54	2393.32	5.7×
Dap(GO2)^7,19,23,29,34^ LL-37	5078.86	120.4	3026.32	not detected *	0.9×
Dap(O1)^8,10,12,15,18,25^ LL-37	4847.41	242.31	2832.34	2360.38	1.9×
Dap(O2)^8,10,12,15,18,25^ LL-37	5115.05	306.21	3052.59	2492.53	2.4×
Dap(MO1)^13,20,24^ LL-37	4760.41	16.47	2743.40	2389.16	0.13×
Dap(MO2)^13,20,24^ LL-37	4894.99	44.41	2831.50	2477.58	0.34×

Half-lives (T½) and major fragment masses determined by LC-MS; fold-change relative to native LL-37. T½ values are single determinations derived from the monoexponential decay fit of the source data; replicate runs were not available in the source dataset, so per-value standard deviations are not reported. Fragment masses are single LC-MS measurements. * For the Dap(GO2)^7,19,23,29,34^ LL-37 analog, only one hydrolysis fragment was observed in solution above the LC-MS detection threshold; see Section 2.3.

**Table 3:** Molecular mass evolution during incubation with PAD2 or PAD4.

Enzyme/Compound	t = 0 h	t = 2 h	t = 4 h	t = 6 h	t = 8 h	t = 24 h
PAD2/LL-37	4491.3	4494.3	4494.3	4495.3	4495.3	4495.3
PAD2/Dap(GO1)^7,19,23,29,34^ LL-37	4858.60	4858.60	4859.60	4859.60	4860.60	4860.60
PAD2/Dap(GO2)^7,19,23,29,34^ LL-37	5078.60	5078.60	5078.60	5079.60	5079.60	5079.60
PAD4/LL-37	4491.3	4494.3	4494.3	4495.3	4495.3	4495.3
PAD4/Dap(GO1)^7,19,23,29,34^ LL-37	4858.60	4858.60	4859.60	4859.60	4860.60	4860.60
PAD4/Dap(GO2)^7,19,23,29,34^ LL-37	5078.60	5078.60	5078.60	5079.60	5079.60	5079.60

Each +1 Da increment corresponds to one Arg → Cit conversion. Masses in Da; values are single LC-MS determinations (molecular mass of the principal species at each time point). Standard deviations were not computed for this single-determination LC-MS time course; replicate runs were not available in the source dataset. PAD2 and PAD4 produced identical mass trajectories within instrument resolution.

**Table 4:** Antibacterial activity (MIC) against three reference strains.

Compound	*E. coli* ATCC 25922 MIC [µM]	*S. aureus* ATCC 25923 MIC [µM]	*P. aeruginosa* ATCC 27853 MIC [µM]	Range Tested
LL-37	4	8	4	1–64 µM
Dap(GO1)^7,19,23,29,34^ LL-37	>64	>64	>64	1–64 µM
Dap(GO2)^7,19,23,29,34^ LL-37	>64	>64	>64	1–64 µM
Dap(O1)^8,10,12,15,18,25^ LL-37	>64	>64	>64	1–64 µM
Dap(O2)^8,10,12,15,18,25^ LL-37	>64	>64	>64	1–64 µM
Dap(MO1)^13,20,24^ LL-37	>64	>64	>64	1–64 µM
Dap(MO2)^13,20,24^ LL-37	>64	>64	>64	1–64 µM

MIC values for LL-37 are typical of the literature for cation-adjusted Mueller–Hinton broth microdilution (CLSI guidelines). “>64” indicates no growth inhibition at the highest concentration tested; the true MIC for the DAPEG analogs lies above 64 µM. *n* = 3 biological replicates with technical duplicates per condition.

**Table 5:** MTT cytotoxicity at 24 h, summarized as approximate IC_50_ (µM) for each compound and cell line.

Compound	HDFa IC_50_	HB2 IC_50_	CRL-1472 IC_50_	HL-60 IC_50_	HL-60 (diff.) IC_50_
LL-37	≈50	≈50	≈50	≈50	>50; ↑ viability
Dap(GO1)^7,19,23,29,34^ LL-37	≈50	≈50	≈50	>50	>50; ↑ viability
Dap(GO2)^7,19,23,29,34^ LL-37	≈50	≈50	≈50	>50	>50; ↑ viability
Dap(O1)^8,10,12,15,18,25^ LL-37	>50	≈50	≈50	>50	>50
Dap(O2)^8,10,12,15,18,25^ LL-37	>50	≈50	≈50	>50	>50
Dap(MO1)^13,20,24^ LL-37	>50	≈50	≈50	>50	>50; mod. suppress.
Dap(MO2)^13,20,24^ LL-37	>50	≈50	≈50	>50	>50; mod. suppress.

IC_50_ values are estimated from the highest concentration showing >50% viability reduction vs. untreated control. “≈50” indicates significant viability reduction first detected at 50 µM (no significant reduction at 20 µM). “>50” indicates no significant viability reduction at the highest tested concentration. “↑ viability”: viability increase relative to untreated control. “mod. suppress.”: modest, non-significant reduction at 50 µM. These categorical assignments are corroborated by the full numerical mean ± SD viability values at all concentrations (1, 5, 10, 20 and 50 µM) for all compounds and cell lines, provided in Supplementary Table S2. *n* = 3 independent experiments; one-way ANOVA with Dunnett’s post hoc test vs. untreated control (* *p* < 0.05, ** *p* < 0.01, *** *p* < 0.001). At 50 µM, both HB2 mammary epithelial and CRL-1472 bladder carcinoma cells showed a significant viability reduction (≈50% viability, IC_50_ ≈ 50 µM) for LL-37 and all six analogs.

**Table 6:** Summary of HL-60 modulation across PR3 and MPO degranulation markers, with and without LPS challenge.

Compound	PR3—Alone	PR3 + LPS	MPO—Alone	MPO + LPS
LL-37	activates	activates synergistically	activates	activates synergistically
Dap(GO1)^7,19,23,29,34^ LL-37	activates	activates synergistically	activates	activates synergistically
Dap(GO2)^7,19,23,29,34^ LL-37	activates	activates synergistically	activates	activates synergistically
Dap(O1)^8,10,12,15,18,25^ LL-37	no activation	suppressed vs. LL-37 + LPS	no activation	suppressed vs. LL-37 + LPS
Dap(O2)^8,10,12,15,18,25^ LL-37	no activation	suppressed vs. LL-37 + LPS	no activation	suppressed vs. LL-37 + LPS
Dap(MO1)^13,20,24^ LL-37	mild activation	partial suppression	mild activation	partial suppression
Dap(MO2)^13,20,24^ LL-37	mild activation	partial suppression	mild activation	partial suppression

Concordant patterns between the two markers support the interpretation of genuine degranulation modulation rather than enzyme-specific effects.

## Data Availability

The data presented in this study are available on request from the corresponding author.

## Supplementary Materials

The following supporting information can be downloaded at https://www.mdpi.com/article/10.3390/ph19081152/s1. Figure S1: PR3 proteolysis kinetics over 24 h, monitored by LC-MS. Each panel shows intact peptide (solid line) and hydrolysis fragments (dotted/dashed); T½ values are shown above each panel. Panels (A–G) correspond to native LL-37 and the six DAPEG analogs, in the order LL-37 (A), Dap(O1) (B), Dap(O2) (C), Dap(GO1) (D), Dap(GO2) (E), Dap(MO1) (F), and Dap(MO2) (G); panel (G) shows Dap(MO2)^13,20,24^ LL-37 (T½ = 44.4 min). Table S1: Cell lines used in cytotoxicity and degranulation assays, with culture media and seeding densities; Table S2: Cell viability (% of untreated control, mean ± SD) at all tested concentrations for LL-37 and the six DAPEG analogs across five cell lines; Table S3: Hydrodynamic size and polydispersity index (PDI) of LL-37 and the six DAPEG analogs alone and in complexes with pUC19 plasmid or 76-bp double-stranded DNA.
