# Supplementary material for: Building Blocks of 2,3-Diaminopropionic Acid Functionalized with an Oxa-Acid Spacer Shift the Functional Profile of Human Cathelicidin LL-37: Enhanced Enzymatic Stability and Selective Suppression of Neutrophil Degranulation
Source: Pharmaceuticals (Basel). 2026 Jul 24;19(8):1152. doi: 10.3390/ph19081152 (PMC13516444; doi:10.3390/ph19081152)
Supplement: Supplementary file 1 [file pharmaceuticals-19-01152-s001.zip › pharmaceuticals-4369139-supplementary.pdf]

Supplementary Information

Building blocks of 2,3-diaminopropionic acid functionalized with an oxa-acid spacer shift the functional profile of human cathelicidin LL-37: enhanced enzymatic stability and selective suppression of neutrophil degranulation

Wiktoria Rejmak, Katarzyna Bury, Wojciech Kamysz, Marta Bauer, Magdalena Wysocka, Adam Lesner

Contents: Table S1 (cell lines and culture media); Table S2 (MTT viability data); Table S3 (DLS hydrodynamic sizes and polydispersity); Figure S1 (PR3 proteolysis kinetics).

Table S1. Cell lines and culture conditions

Table S1. *Cell lines used in cytotoxicity and degranulation assays, with culture media and seeding densities.*

Cell line	Description	Culture medium	Seeding density ($\times 10^3$ cells/well)
HB2	Human breast epithelial cells	DMEM HG + 10% heat-inactivated FBS + 1% penicillin/streptomycin + 5 µg/mL insulin + 5 µg/mL hydrocortisone	5
CRL-1472	Human bladder transitional carcinoma, stage III	DMEM HG + 10% heat-inactivated FBS + 1% penicillin/streptomycin	8
HDFa	Human primary dermal fibroblasts	DMEM + 10% heat-inactivated FBS + 1% penicillin/streptomycin	4
HL-60	Human acute promyelocytic leukemia (suspension)	RPMI-1640 + 10% heat-inactivated FBS + 1% penicillin/streptomycin	25.5
HL-60 (differentiated)	HL-60 differentiated toward a neutrophil-like phenotype	RPMI-1640 + 10% FBS + 1% antibiotics + 1.25% DMSO, 5 days, daily medium exchange, cell count maintained at 10×10^6	see Methods 2.9

Culture conditions: 37 °C, 5% CO_2. FBS, fetal bovine serum; DMEM HG, Dulbecco's Modified Eagle Medium high glucose; DMSO, dimethyl sulfoxide. .

Table S2. MTT cell-viability data

Table S2. *Cell viability (% of untreated control, mean ± SD) at all tested concentrations for LL-37 and the six DAPEG analogs across five cell lines.*

Compound	Conc. (µM)	HDFa	HB2	CRL-1472	HL-60	HL-60 (diff.)
LL-37	1	100 ± 16	100 ± 14	100 ± 12	100 ± 8	100 ± 15
	5	110 ± 21	104 ± 17	118 ± 11	112 ± 17	105 ± 21
	10	95 ± 17	92 ± 13	108 ± 10	125 ± 21	110 ± 11
	20	97 ± 13	110 ± 18	109 ± 14	114 ± 13	115 ± 12
	50	42 ± 13**	37 ± 11***	31 ± 6***	42 ± 7**	118 ± 10
Dap(GO1)[7,19,23,29,34] LL-37	1	102 ± 11	99 ± 13	101 ± 9	98 ± 7	102 ± 12
	5	98 ± 14	103 ± 10	97 ± 12	101 ± 11	106 ± 14
	10	101 ± 12	96 ± 11	104 ± 8	96 ± 14	112 ± 9
	20	95 ± 9	98 ± 14	98 ± 11	104 ± 10	119 ± 13
	50	48 ± 13***	44 ± 12***	41 ± 7***	97 ± 9	124 ± 11
Dap(GO2)[7,19,23,29,34] LL-37	1	99 ± 10	101 ± 12	98 ± 14	102 ± 8	101 ± 11
	5	104 ± 13	97 ± 9	102 ± 11	99 ± 13	108 ± 15
	10	97 ± 11	102 ± 14	95 ± 10	105 ± 11	115 ± 10
	20	96 ± 8	94 ± 11	99 ± 12	98 ± 12	122 ± 14
	50	51 ± 12***	46 ± 10***	45 ± 8***	101 ± 14	128 ± 13
Dap(O1)[8,10,12,15,18,25] LL-37	1	101 ± 12	98 ± 11	103 ± 13	97 ± 6	99 ± 12
	5	103 ± 9	101 ± 14	99 ± 10	102 ± 11	103 ± 14
	10	98 ± 14	96 ± 8	101 ± 11	96 ± 13	98 ± 10
	20	102 ± 11	95 ± 12	96 ± 9	101 ± 10	104 ± 11
	50	94 ± 13	53 ± 11**	49 ± 7***	96 ± 12	98 ± 15
Dap(O2)[8,10,12,15,18,25] LL-37	1	100 ± 11	102 ± 13	99 ± 9	101 ± 9	102 ± 11
	5	97 ± 13	98 ± 10	104 ± 12	104 ± 12	101 ± 13
	10	104 ± 10	101 ± 12	97 ± 11	98 ± 14	97 ± 8
	20	101 ± 14	97 ± 9	102 ± 13	99 ± 11	105 ± 12
	50	96 ± 12	48 ± 10***	46 ± 6***	103 ± 13	102 ± 14
Dap(MO1)[13,20,24] LL-37	1	102 ± 12	99 ± 11	101 ± 14	99 ± 8	100 ± 13
	5	99 ± 14	102 ± 13	96 ± 11	103 ± 11	104 ± 11
	10	103 ± 11	97 ± 9	104 ± 10	96 ± 12	98 ± 9
	20	98 ± 9	99 ± 12	98 ± 12	101 ± 10	103 ± 12
	50	95 ± 10	52 ± 14**	47 ± 8***	97 ± 13	86 ± 11
Dap(MO2)[13,20,24] LL-37	1	98 ± 10	101 ± 14	102 ± 12	102 ± 7	103 ± 11
	5	101 ± 13	96 ± 10	98 ± 11	98 ± 13	99 ± 14
	10	96 ± 12	104 ± 12	101 ± 9	104 ± 11	105 ± 10
	20	104 ± 11	98 ± 13	95 ± 14	99 ± 9	101 ± 12
	50	93 ± 14	49 ± 11**	44 ± 7***	102 ± 12	84 ± 13

*All values are mean ± SD of n = 3 independent MTT experiments (three technical replicates each), expressed as percentage of the untreated control. Concentrations tested: 1, 5, 10, 20 and 50 µM. Statistical significance versus the untreated control was assessed by one-way ANOVA with Dunnett's post hoc test; significance is indicated per cell as * p < 0.05, ** p < 0.01, *** p < 0.001 (ns, not*

significant). The same dataset underlies main-text Table 5; cell-line column order matches that table. Significance markers denote a significant reduction relative to the untreated control; cells without a marker did not differ significantly from control (ns).

Table S3. Dynamic light scattering (DLS) data

Table S3. *Hydrodynamic size and polydispersity index (PDI) of LL-37 and the six DAPEG analogs alone and in complexes with pUC19 plasmid or 76-bp double-stranded DNA.*

Compound	Alone — diameter [nm]	Alone — PDI [%]	+ pUC19 — diameter [nm]	+ pUC19 — PDI [%]	+ dsDNA — diameter [nm]	+ dsDNA — PDI [%]
pUC19 (alone)	—	—	141.72 ± 7.24	21.46 ± 2.65	—	—
76-bp dsDNA (alone)	—	—	—	—	584.10 ± 14.25	29.43 ± 3.12
LL-37	208.03 ± 12.66	20.42 ± 1.41	8.55 ± 0.50	23.56 ± 2.68	63.79 ± 4.81	21.48 ± 2.14
Dap(GO1)[7,19,23,29,34] LL-37	202.15 ± 6.40	25.48 ± 2.98	68.58 ± 4.73	23.41 ± 1.78	54.66 ± 6.56	25.14 ± 1.85
Dap(GO2)[7,19,23,29,34] LL-37	199.92 ± 1.96	27.78 ± 1.51	76.01 ± 9.01	26.60 ± 1.11	92.01 ± 4.78	26.42 ± 2.16
Dap(MO1)[13,20,24] LL-37	187.33 ± 7.35	23.46 ± 2.39	115.83 ± 10.37	26.48 ± 3.10	368.04 ± 14.33	22.65 ± 3.01
Dap(MO2)[13,20,24] LL-37	127.09 ± 9.62	24.17 ± 2.29	172.51 ± 8.78	22.12 ± 1.06	364.51 ± 9.88	19.68 ± 1.45
Dap(O1)[8,10,12,15,18,25] LL-37	159.73 ± 17.23	22.68 ± 1.29	78.19 ± 3.58	26.80 ± 1.13	333.73 ± 6.60	23.58 ± 2.36
Dap(O2)[8,10,12,15,18,25] LL-37	213.68 ± 8.59	26.45 ± 1.68	81.13 ± 14.21	18.58 ± 1.48	303.05 ± 12.94	19.69 ± 1.87

Values are mean ± SD hydrodynamic diameters of n = 3 independent DLS measurements; PDI, polydispersity index.

Supplementary Figure

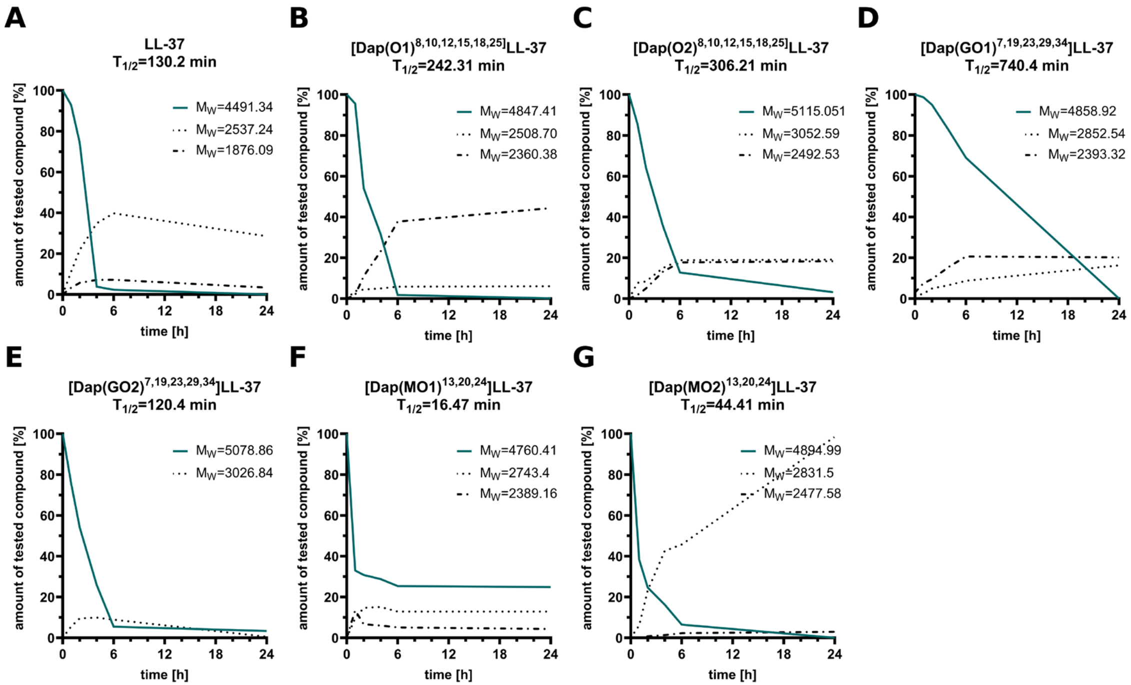

Figure S1. PR3 proteolysis kinetics over 24 h, monitored by LC-MS. Each panel shows intact peptide (solid line) and hydrolysis fragments (dotted/dashed); T½ values are shown above each panel. Panels A–G correspond to native LL-37 and the six DAPEG analogs, in the order LL-37 (A), Dap(O1) (B), Dap(O2) (C), Dap(GO1) (D), Dap(GO2) (E), Dap(MO1) (F), and Dap(MO2) (G); panel G shows Dap(MO2)^{13,20,24} LL-37 (T½ = 44.4 min).
